# Editorial: Neuroscience, computing, performance, and benchmarks: Why it matters to neuroscience how fast we can compute

**DOI:** 10.3389/fninf.2023.1157418

**Published:** 2023-03-30

**Authors:** James B. Aimone, Omar Awile, Markus Diesmann, James C. Knight, Thomas Nowotny, Felix Schürmann

**Affiliations:** ^1^Neural Exploration and Research Laboratory, Center for Computing Research, Sandia National Laboratories, Albuquerque, NM, United States; ^2^Blue Brain Project, École Polytechnique Fédérale de Lausanne, Geneva, Switzerland; ^3^Institute of Neuroscience and Medicine and Institute for Advanced Simulation and JARA-Institute Brain Structure-Function Relationships, Jülich Research Centre, Jülich, Germany; ^4^Department of Physics, Faculty 1, RWTH Aachen University, Aachen, Germany; ^5^Department of Psychiatry, Psychotherapy and Psychosomatics, School of Medicine, RWTH Aachen University, Aachen, Germany; ^6^School of Engineering and Informatics, University of Sussex, Brighton, United Kingdom

**Keywords:** performance benchmarking, large-scale simulation, simulation workflows, software sustainability, neuromorphic computing architectures, subcellular neurosimulation, biophysically detailed models

## Introduction

At the turn of the millennium the computational neuroscience community realized that neuroscience was in a software crisis: software development was no longer progressing as expected and reproducibility declined. The International Neuroinformatics Coordinating Facility (INCF) was inaugurated in 2007 as an initiative to improve this situation. The INCF has since pursued its mission to help the development of standards and best practices. In a community paper published this very same year, Brette et al. (2007) tried to assess the state of the field and to establish a scientific approach to simulation technology, addressing foundational topics, such as which simulation schemes are best suited for the types of models we see in neuroscience.

In 2015, a Frontiers Research Topic “*Python in neuroscience*” by Muller et al. (2015) triggered and documented a revolution in the neuroscience community, namely in the usage of the scripting language Python as a common language for interfacing with simulation codes and connecting between applications. The review by Einevoll et al. (2019) documented that simulation tools have since further matured and become reliable research instruments used by many scientific groups for their respective questions. Open source and community standard simulators today allow research groups to focus on their scientific questions and leave the details of the computational work to the community of simulator developers.

A parallel development has occurred, which has been barely visible in neuroscientific circles beyond the community of simulator developers: Supercomputers used for large and complex scientific calculations have increased their performance from ~10 TeraFLOPS (10^13^ floating point operations per second) in the early 2000s to above 1 ExaFLOPS (10^18^ floating point operations per second) in the year 2022. This represents a 100,000-fold increase in our computational capabilities, or almost 17 doublings of computational capability in 22 years. Moore's law (the observation that it is economically viable to double the number of transistors in an integrated circuit every other 18–24 months) explains a part of this; our ability and willingness to build and operate physically larger computers, explains another part. It should be clear, however, that such a technological advancement requires software adaptations and under the hood, simulators had to reinvent themselves and change substantially to embrace this technological opportunity. It actually is quite remarkable that—apart from the change in semantics for the parallelization—this has mostly happened without the users knowing.

The current Research Topic was motivated by the wish to assemble an update on the state of neuroscientific software (mostly simulators) in 2022, to assess whether we can see more clearly which scientific questions can (or cannot) be asked due to our increased capability of simulation, and also to anticipate whether and for how long we can expect this increase of computational capabilities to continue.

## Larger brain and brain tissue models

A promising advance compared to the state of the field 15 years ago is that we now see an increase in the complexity of network models. Earlier, the balanced random network model composed of a population of excitatory neurons and a population of inhibitory neurons was dominating the literature and few studies reached beyond it. Today, biologically much more realistic network models are in widespread use and have become the new *de facto* standard (Albers et al.; Tiddia et al.; Awile et al.; Borges et al.). These newer models represent the anatomy of the local circuitry of the mammalian cortex at full scale, meaning with all the neurons and synapses. As a consequence, neuron and synapse numbers have increased by an order of magnitude compared to earlier models. The ability to simulate at full scale is decisive because this removes all uncertainties on the scaling of emerging network phenomena with network size which have plagued and occupied theoreticians for a long time (van Albada et al., 2015).

## Expansion to the subcellular realm

Most articles in this collection concentrate on describing models developed at the level of neurons and synapses. However, some articles also show how our advances in computing and simulation technology can be used to extend our modeling and simulation capability to the membrane and subcellular biochemical realm. Awile et al. show how subcellular dynamics can be integrated into NEURON simulations. The works of Chen et al. and McDougal et al. enable neuroscientists to study the biophysics of synaptic plasticity and the processes in the spine in detail. As generally accepted models of plastic processes have not yet been established on a phenomenological level, the capability to simulate on the level of subcellular processes is of high relevance.

## The role of simulators and workflows

The number of codes targeting the same level of description has decreased somewhat and remaining codes like NEURON (Awile et al.) and NEST (Albers et al.; Pronold et al.) have increasingly embraced and advanced community-based development models and incorporated ideas of the emerging field of research software engineering (RSE). At the same time, it is remarkable that after 15 years of intense research the seemingly fundamental question of whether an event-driven or a clock-driven approach to the simulation of spiking neuronal networks is more efficient, does not seem to have found a consensus (Mo and Tao; Hanuschkin et al., 2010; Krishnan et al., 2018). A reason for this could of course be that there is simply no general answer for any model and hardware, and that in practice simulation codes such as NEURON and NEST employ hybrid approaches.

Furthermore, various variants of language interfaces were developed for the traditional simulation codes (Borges et al.; Herbers et al.). Also new simulation codes were developed expressing network models entirely in Python or implementing code generators for performance critical sections (Dinkelbach et al.; Alevi et al.). Of similar importance to the advances of individual tools is the progress in the digitalization of scientific workflows (Albers et al.; Awile et al.; Feldotto et al.; Herbers et al.) and the observation that not only the source codes but also executable model descriptions of simulation engines are available in publicly curated repositories.

## Keeping innovations around—Sustainability of scientific software

Software codes that have been around for 15 years, are still in widespread use by the community today. Neuroscience must therefore acknowledge, as other scientific fields already have, that scientific software can easily have life spans of 40 years or more. Sustainability and portability are consequently of high relevance for software tools that serve a whole community rather than a specific scientific goal as showcased in Chen et al. and Awile et al.. While often new features or increased performance (especially in the case of simulators) are the milestones of such projects, the authors observed that a focus on software sustainability can be an important driver for innovations. Both publications show how the modernization of complex scientific software can be made more tractable by first focusing on putting in place a robust continuous integration, testing, and documentation workflow. As the software developed in the field is becoming more complex to satisfy the scientific needs (e.g., supporting multiple numerical methods, multiphysics simulations, and heterogenous hardware platforms), the implementation of software modularity and composability is concurrently becoming increasingly important. These methodologies feature prominently in Feldotto et al.. The authors focus here on container technologies to enable complex software setups and workflows for embodied simulations of spiking neural networks.

## If simulator engines are on track, how about analysis packages?

Only one paper in this series discusses the performance of a data analytics problem (Porrmann et al.). This may reflect the possibility that the availability of HPC methods is not the most pressing problem in the analysis of neuroscientific data. There is certainly considerable activity in processing pipelines for neuroimaging, but this field finds other forums (Halchenko et al., 2021). Maybe the discrepancy also reflects the fact that in the research field concerned with the spiking activity of neuronal networks, researchers doing simulations have always been somewhat advanced in embracing new hardware and software technologies compared to those involved in analysis.

## Embracing the course of computing architecture evolution

A thread running through many of the articles in this collection is how to make the best of the currently available but rapidly changing hardware systems. Since clock frequencies for processors flattened out in the mid-2000s, processor architectures have become progressively more parallel. This applies to latency-optimized CPUs which have become moderately parallel (<100 superscalar cores/CPU) as well as GPUs (>1000s of simple cores/GPU). It is heartening to see that the community is embracing this opportunity and challenge. Alevi et al. present new software for exploiting NVIDIA GPU hardware to accelerate simulation with the popular Brian simulator (Stimberg et al., 2019), complementing the existing Brian2GeNN software (Stimberg et al., 2020). Awile et al. show how code generation can be used to run the NEURON simulator on GPUs. In a similar vein, Tiddia et al. present work on how to efficiently run a large spiking neural network model on a GPU cluster and Dinkelbach et al. describe work on one specific aspect of efficient simulations of spiking neural networks on GPU hardware in their ANNarchy simulation software. Ladd et al. furthermore present an evolutionary algorithm able to run on GPUs that accelerates the building of multi-compartment neuron models. Challenges of how to handle massive parallelism and distributed computing also arise in the context of classical HPC clusters, and Pronold et al. describe how one key bottleneck can be overcome.

## Emerging computing architectures

The unsure future of CMOS scaling will present the neural simulation community with an even broader set of architectures beyond CPUs and GPUs. There is an increasing trend toward more specialized components, particularly those that enable artificial intelligence applications such as artificial neural networks (Reed et al., 2022). We hope that such specialization may also enable simulations of biological neural networks without too many adaptations. Looking beyond ANN accelerators, it is also reasonable to expect to see even more diversity through platforms, such as neuromorphic hardware, obtaining widespread use in HPC systems, particularly since they are proving suitable for conventional computing applications (Aimone et al., 2022). Beyond exploiting specific characteristics of biological neural networks, today's neuromorphic computing systems such as SpiNNaker, BrainScales, and Loihi attempt an integration at scale. As a result they enable complex models to be programmed, with biologically fit neurons shown to be realizable on Intel Loihi (Dey and Dimitrov), BrainScaleS-2 (Müller et al.), and SpiNNaker (Peres and Rhodes; Ward and Rhodes).

## Rethinking the underlying algorithms

Not only is the computational neuroscience community embracing the challenges of rapidly developing processor architectures but it is also capitalizing on the additional computing power to explore different simulation algorithms and schemes. For instance, Osborne and de Kamps extend the population density technique for neural network simulations to higher-dimensional neuron models and Chen et al. improve on memory efficiency and simulation speed for detailed molecular simulations of neurons. Similarly, McDougal et al. describe the efficient simulation of 3D reaction-diffusion processes in neuronal networks extending on more traditional 1D simulations for dendrites and axons.

## Time

While GPUs and large, massively-parallel HPC clusters were not built for the purpose of brain simulations, the inherently parallel nature of how brains operate, makes such systems reasonably well-suited to simulating brain models. However, we must not forget that while computers have become more powerful (i.e., they are able to do more things in parallel), they have not become much faster—ever since frequency scaling (Dennard Scaling) had to stop due to limits in how much heat can be dissipated from an integrated circuit. This puts in question certain scientific problems which require the simulation of long time durations such as needed, for example, in plasticity studies, or extensive training runs in the emerging field of neuro-inspired machine learning. While algorithmic innovations may help us to rethink the supposedly critical sequential paths of computational problems (e.g., AlphaFold applied these to the problem of protein folding), an alternative approach may be the acceleration factors that can be achieved from mapping the computational problem to physical instantiations of the computation such as done by Brainscales-2 (Müller et al.) or as indicated by Trensch and Morrison through spatial computations using SoCs and FPGAs.

## Benchmarking as the compass

As the diversity of hardware architectures grows, it will be increasingly important to quantify the suitability of those platforms for actual brain tissue model simulations. It is thus necessary to develop benchmarks (models) and benchmarking (measuring) to objectively quantify the performance of such platforms. While HPC systems have often varied in components and configurations, there have long been standards for linear algebra such as Linpack that allowed rigorous, even if not perfect, comparisons. Herbers et al., Albers et al., and Schmitt et al. make a step toward generic and simulator agnostic frameworks for benchmarking and simulation. However, as we look toward a future with specialized neural network accelerators and general purpose von Neumann systems, the challenge in benchmarking will become more pronounced. This is especially a challenge with neuromorphic hardware, which is both rapidly evolving and exhibits a diversity of approaches with mixed advantages in speed and energy, resulting in a complex basis for evaluation (Trensch and Morrison; Müller et al.). Furthermore, the concept of a FLOP or matrix multiply operation is less meaningful in spiking neural simulations which may be event-driven and sparse. One proposed approach is to develop concrete benchmark spiking networks that can be tested on both neuromorphic systems and conventional processors, which is proving useful in obtaining an early assessment of the relative efficiency of neuromorphic systems compared to both conventional systems and real brains (Ostrau et al.; Kurth et al., 2022).

## Author contributions

All authors contributed equally to the editing of the Research Topic. All authors contributed equally to the writing of the article and approved the submitted version.
